# Opossum Mammary Maturation as It Relates to Immune Cell Infiltration and Nutritional Gene Transcription

**DOI:** 10.1093/iob/obz036

**Published:** 2019-12-30

**Authors:** B D Fehrenkamp, R D Miller

**Affiliations:** Center for Evolutionary and Theoretical Immunology, Biology Department, University of New Mexico, UNM Biology, Castetter Hall 1480, MSC03-2020, 219 Yale Blvd NE, Albuquerque, NM 87131-0001, USA

## Abstract

The mammary gland has evolved to accommodate the developmental needs of offspring in species-specific ways. This is particularly true for marsupials. Marsupial milk content changes dramatically throughout lactation in ways appearing timed with neonatal ontogeny and behavior. Here we investigate morphological restructuring within the mammaries throughout lactation in the gray short-tailed opossum, *Monodelphis domestica*. Substantial remodeling of the mammaries occurs throughout the first half of active lactation. It is not until the latter half of lactation that opossum mammaries appear histologically similar to active eutherian mammaries. Noteworthy was the presence of eosinophils in early developing mammary tissue, which correlated with elevated abundance of transcripts encoding the chemokine IL-16. The presence and abundance of whey protein transcripts within the opossum mammaries were also quantified. Whey acidic protein (WAP) transcript abundance peaked in the latter half of lactation and remained elevated through weaning. Minimal transcripts for the marsupial-specific Early and Late Lactation Proteins (ELP/LLP) were detected during active lactation. Elevated abundance of LLP transcripts was only detected prior to parturition. Overall, the results support the role of eosinophils in mammary restructuring appearing early in mammalian evolution, and describe key similarities and differences in nutritional protein transcript abundance among marsupial species.

## Introduction

A defining trait of mammals is the presence of a specialized mammary gland for milk production. This is found in all three extant mammalian lineages: Prototheria, Metatheria, and Eutheria (Lefevre et al. 2010). Prototheria is the only oviparous lineage. The mammary is a dynamic tissue that undergoes substantial remodeling for milk production. In eutherian species investigated, remodeling occurs during pregnancy in preparation for lactogenesis. This includes an increase in secondary and tertiary ductal branching, providing the network for the development of the alveoli, where milk synthesis and storage occurs (Djonov et al. 2001; Macias and Hinck 2012). Proliferating epithelial cells generate alveolar buds that progressively cleave and differentiate into distinct alveoli, which become milk-secreting lobules during lactation (Macias and Hinck 2012). Interstitial adipose tissue recedes as the proliferating epithelial cells occupy the interductal spaces. Increased vascularization occurs and by mid-pregnancy, and each alveoli is surrounded by a basket-like network of capillaries. By late pregnancy the alveoli encompass the majority of the fat pad and some secretory activity has been noted as pregnancy approaches term (Djonov et al. 2001).

There is a growing body of evidence of the role of cells of the immune system in the development and remodeling of mammary tissue (reviewed in Reed and Schwertfeger 2010). Eosinophils in particular appear to participate in eutherian mammary development and remodeling during puberty as well as pregnancy (reviewed in Reed and Schwertfeger 2010). Most eutherian mammary development occurs during pregnancy and by parturition appears complete and there is little change in architecture until involution at the termination of lactation (Djonov et al. 2001; Macias and Hinck 2012).

Comparative studies can provide insights into the origins and conserved features of mammary development. A key group of mammals for comparison are the Metatheria or marsupials, a lineage that diverged from eutherians approximately 165 million years ago (Bininda-Emonds et al. 2007). Marsupials give birth to altricial young that complete developmental stages resembling eutherian fetal development outside of the womb, sometimes in a pouch (Tyndale-Biscoe and Janssens 1988). Marsupials provide an important contrast to what is known regarding eutherian mammary development. Marsupials and eutherians differ in many ways, including the contribution the placenta provides the fetus. Indeed, many of the developmental stages that occur in the prenatal eutherian are postnatal in marsupials. For example, major systems like respiratory, excretory, and even components of the adaptive immune system develop postnatally in the opossum (Yadav et al. 1972; Basden et al. 1997; Buaboocha and Gemmell 1997; Parra et al. 2009; Wang et al. 2012). Marsupials transition from dependence on the placenta for nutrition to dependence on milk earlier in their ontogeny than eutherians (Tyndale-Biscoe and Renfree 1987). However, little is known regarding marsupial mammary structure or development.

Gross milk composition varies across species. All marsupial species studied to date display similar changes in macromolecule composition across lactation, in which early milk is dilute and comprised of mainly carbohydrates; later in lactation milk is of high protein and fat concentrations (Green et al. 1991). Traditionally marsupial lactation has been described using relative macromolecule concentrations as well as whey protein expression (Tyndale-Biscoe and Janssens 1988; Joss et al. 2009). Whey acidic protein (WAP) is a major whey protein and one of the most studied across the lineages. Two milk proteins unique to marsupials are early lactation protein (ELP) and late lactation protein (LLP) (Nicholas et al. 1987; Joss et al. 2009). Although their functions remain unknown they have been used to define changes in milk composition in Australasian marsupials (Kuy et al. 2007; Joss et al. 2009). Studies in Australian species have identified two paralogs of LLP, called LLP-A and LLP-B (Trott et al. 2002; Joss et al. 2009; Hewavisenti et al. 2016). The *Monodelphis domestica* genome contains a single gene for ELP and a single gene for LLP, which appears orthologous to LLP-B. LLP-A has not been identified in non-Australian marsupials (Hewavisenti et al. 2016).

To gain a greater understanding of the evolution of the development of marsupial mammary tissue we investigated mammary architecture in a model marsupial, the gray-short tailed opossum, *M. domestica. M*. *domestica* has been utilized as a laboratory model species for several decades. It is a small (80–120 g), pouch less, nocturnal, omnivore opossum native to South America, specifically Brazil and surrounding countries (VandeBerg and Williams-Blangero 2010). This species is arguably one of the better marsupial models and has a sequenced and well-annotated genome (Mikkelsen et al. 2007). The opossum has a short gestation and extended lactation period. Here we describe the changes in mammary architecture throughout the lactation period with correlation to changes in immune cell composition and key nutritional gene transcript abundance. Marsupial specific nutritional gene transcript abundance is also compared among Australian and American marsupials.

## Materials and methods

### Animals and tissue collection

*M* *onodelphis* *domestica* used were from a captive-bred research colony housed at the University of New Mexico Department of Biology Animal Research Facility. Animals were euthanized by inhaled isoflurane overdose until no evidence of breathing or heartbeat for 1 min, followed by decapitation. This study was approved under protocol numbers 16-200407-MC and 15-200334-B-MC from the University of New Mexico Institutional Animal Care and Use Committee.

Mammary tissue for RNA isolation was collected from at least three females at each time point. This included the last 24 h of pregnancy (embryonic day [E] 13). For prenatal tissue, pregnancies were timed as previously described (Hansen et al. 2017). In addition, tissues were collected from post-partum (P) days 1, 2, 3, 5, 7, 10, 13, 16, 17, 20, 22, 26, 31, 32, 33, 36, 38, 44, and 52. Post-weaning tissue was collected from mothers 24–48 h after pups had been removed and housed separately at P56 (Supplementary Table S1). The number of previous pregnancies prior to when tissue was collected ranged from 0 to 6 per animal with a median of 2. Tissues were preserved in RNALater buffer (Invitrogen, Carlsbad, CA) at 4°C for 48 h. The buffer was then removed and tissues were stored at −80°C until extraction.

Mammary tissues from E3 and E13, as well as P3, 7, 10, 13, 17, 26, 33, 36, and 44 were also collected and preserved for histology following the methods of Old and Deane (2003). Tissues were preserved in 10% buffered formalin (Sigma Aldrich, St. Louis, MO) at 4°C for 24–48 h, then washed repeatedly in 70% ethanol solutions to remove any residual formalin, before being dehydrated and embedded in paraffin wax. Embedded tissues were sectioned to 6 µ and mounted to Apex Superior Adhesive Glass slides (Leica Biosystems, Wetzlar, Germany).

### Histology and microscopy

For morphological examinations, paraffin embedded mammary sections were Hematoxylin and Eosin (H&E) stained and preserved by covering slipping with DPX (Sigma Aldrich, St. Louis, MO). Single field of view bright field microscopy was performed on an inverted Eclipse Nikon Ti utilizing Nikon ARS (Nikon, Minato, Tokyo, Japan) software. A minimum of 36 mammary sections interspersed throughout the tissue was examined per time point to evaluate morphological changes using previously described characteristics in eutherian mammaries.

### RNA extraction and cDNA synthesis

Whole RNA was extracted from mammary tissues using phenol based extraction methods and the Pure Link RNA mini kit (Invitrogen). Residual DNA was removed using the TURBO DNA-free Kit according to manufacturers’ recommended protocols (Invitrogen). Then, 500 ng of DNA-cleaned RNA was used for cDNA synthesis by reverse transcriptase PCR (RT-PCR) using SuperScript III First Strand Synthesis kit (Invitrogen). To reduce bias generated during reverse transcription, reactions were constructed in triplicate and pooled.

### Quantifying gene transcripts

Transcript abundance of specific genes was assessed by quantitative real-time PCR (qPCR) using Sso Advanced Universal SYBR Green Supermix (BioRad, Hercules, CA) according to manufacturer’s instructions for 20 μL reactions. qPCR was performed in triplicate on a BioRad CFX96. Amplification cycling parameters were an initial denaturation step at 95°C for 2 min, followed by 40 cycles of 95°C for 5 s and annealing temperature (varied, see Supplementary Table S2) for 30 s, a terminating step of 95°C for 5 s terminating at 65°C for 31 s. A final melt curve was constructed by 60 cycles of 65°C for 5 s increasing +0.05°C/cycle with a ramp of 0.05°C/cycle. Singularity of product as well as product size was examined per plate by melt curve analyses. A sample from the serial dilution was run on a 2% agarose gel and stained with RedGel Nucleic Acid Stain and viewed under UV light to confirm that a band of the correct size was amplified.

Primers were designed for the *M*. *domestica* genome according to manufacturer’s parameters for qPCR primers (Supplementary Table S2). Efficiency of the amplification was determined for each primer pair using serial 10-fold dilutions of pooled cDNA. Primer pairs were analyzed for singularity of product by the presence of a single band in qPCR, melt curve analyses, as well as sequencing of qPCR product. Sequenced products had 100% identity and alignment with target sequence (not shown). Transcript abundance was normalized for each individual using the Vandersompele method of incorporating multiple reference gene expression levels (Vandersompele et al. 2002). Appropriateness of reference genes was evaluated by calculating *M*-values and target stability scores across all samples. Reference genes used in this study were succinate dehydrogenase subunit A (*SDHA*) and actin-related protein 2 (*ACTR2*). These genes were highly expressed at all time points with little variance and had *M*-values <1 as defined in Vandersompele et al. (2002).

### Statistical analyses

All statistical analyses were performed using default parameters of Prism 7 software (GraphPad, La Jolla, CA). Normalized abundance was calculated per biological replicate per time point. Mean expression was then calculated per biological set. Grubbs outlier analyses were performed for each biological set. Normalized expression of biological replicates including significant outliers is represented in Supplementary Fig. S1. Inclusion of outliers did not significantly alter statistical analyses. Data, not including outliers, were pooled by week (Supplementary Table S1) and mean normalized abundance and standard errors of the mean (SEM) are reported per gene target.

## Results

**Fig. 1:**
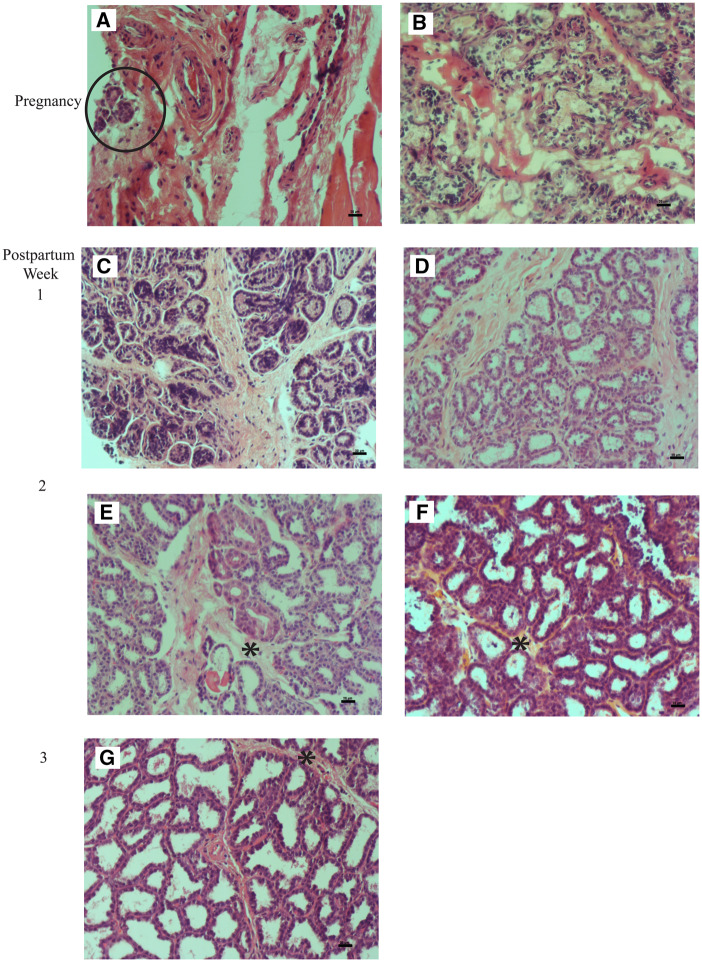
Morphological changes within opossum mammaries throughout early lactation. H&E-stained mammary sections from *pregnancy*, embryonic/gestational (E) day 3 (**A**), and E13 (**B**). Circle indicates beginning alveolar development. Field is predominated with connective and skeletal muscle tissue with minimal alveolar development in pregnancy. Following birth, postnatal/postpartum (P) *week 1*: P3 (**C**), and P7 (**D**), alveoli continue to develop with minimal evidence of ducting present. During *week 2*, P10 (**E**), and P13 (**F**), early ducting structures are present. * designates ducting structures. Secondary ducting structures are evident in *week 3*, P17 (**G**). Images are of 20× magnification. Scale in bottom right corner of each image = 20 µ.

**Fig. 2:**
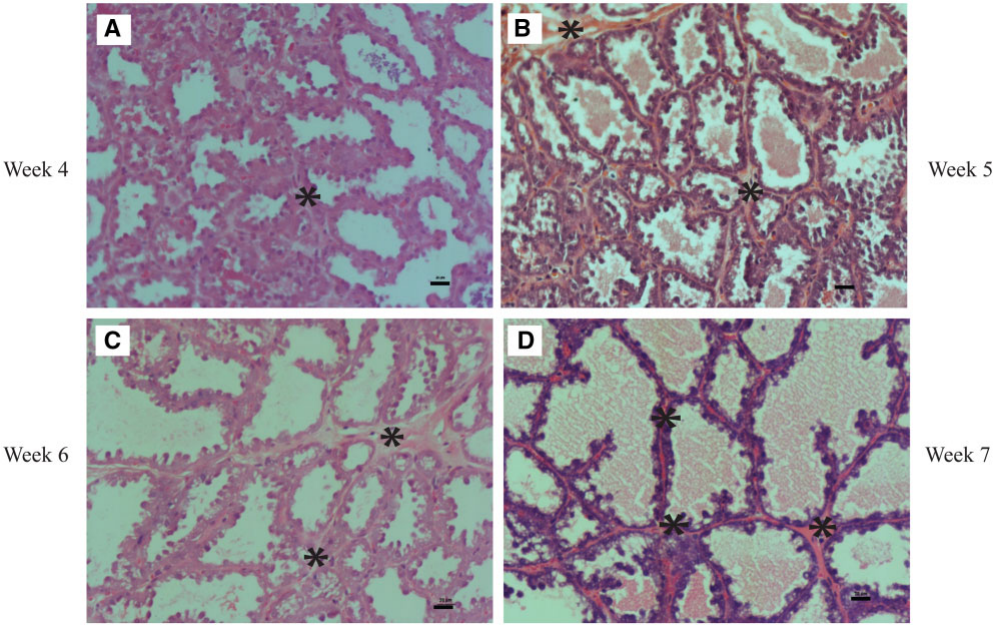
Morphological changes within opossum mammaries throughout later lactation. H&E-stained mammary sections from *week 4*: P26 (**A**), week 5: P32 (**B**), *week 6*: P36 (**C**), and week 7: P44 (**D**). Highly defined alveoli as well as secondary and tertiary ductal branching was evident during the later half of lactation. * indicates ducting structures. By week 5 and beyond alveoli encompass the majority of the fat pad and tissues are morphologically similar to active eutherian mammaries. Images are of 20× magnification. Scale in bottom right corner of each image = 20 µ.

We investigated the morphological changes that opossum mammaries undergo throughout lactation and correlated these with the timing and abundance of specific nutritional gene transcripts. Mammary tissue from three biological replicates per time point, across 11 time points spanning from gestation through the first 7 of the 8 weeks of lactation, were examined for the presence of alveolar development and ductal branching (Figs. 1 and 2, andSupplementary Table S1).

Prior to parturition, opossum mammaries were predominately connective tissue and gland development was minimal with few alveoli present (Fig. 1A, B). Increased alveoli number was evident within the first week of lactation; however, connective tissue still predominated and minimal signs of ducting were present (Fig. 1C, D). By the second week of lactation early duct structures were evident. Alveoli increased both in number and size in week 2 of lactation, compared with earlier time points (Fig. 1E, F**)**. By the third week of lactation alveoli continued to increase in size and secondary ducting was also evident (Fig. 1G).

Cell density within a given field of view decreased throughout opossum lactation, as more of the mammary space was dedicated to milk storage (Figs. 1 and 2). The presence of highly defined alveoli and tertiary ductal branching was not evident until week 4 (Fig. 2A). It was not until week 5 and later, in which the alveoli encompassed the majority of the fat pad and structured ducting was evident throughout the tissue (Fig. 2B–D). Enlarged alveoli and decreased connective tissue were seen weeks 5 and later. Throughout the latter half of lactation, weeks 5 and beyond, opossum mammary tissue appeared developmentally equivalent to active eutherian mammary tissue (Fig. 2B–D). Histological remodeling early in lactation in opossum mammary tissue correlates developmentally to restructuring that occurs during pregnancy in eutherians, prior to milk production (Djonov et al. 2001; Macias and Hinck 2012). An increased maturity in opossum mammary development corresponds with previously reported changes in nutritional composition and quantity of milk produced (Green et al. 1991).

**Fig. 3:**
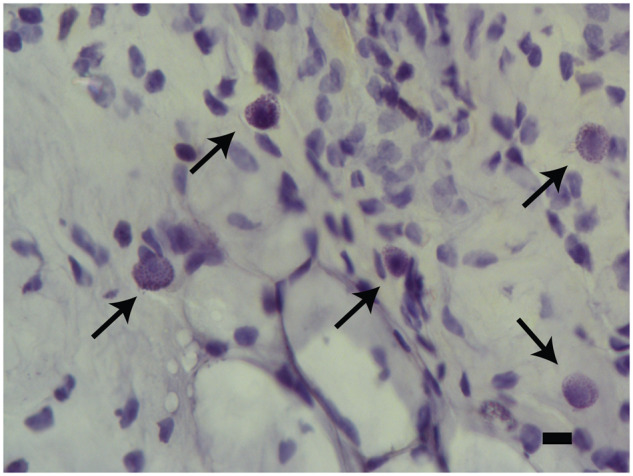
Eosinophil presence within early lactating opossum mammaries. 40× magnification of P3, week 1, mammary tissue stained in hematoxylin. Arrows indicate eosinophils. Eosinophils were detected in the stroma surrounding developing aveoli. Scale in lower right = 10 μm.

**Fig. 4:**
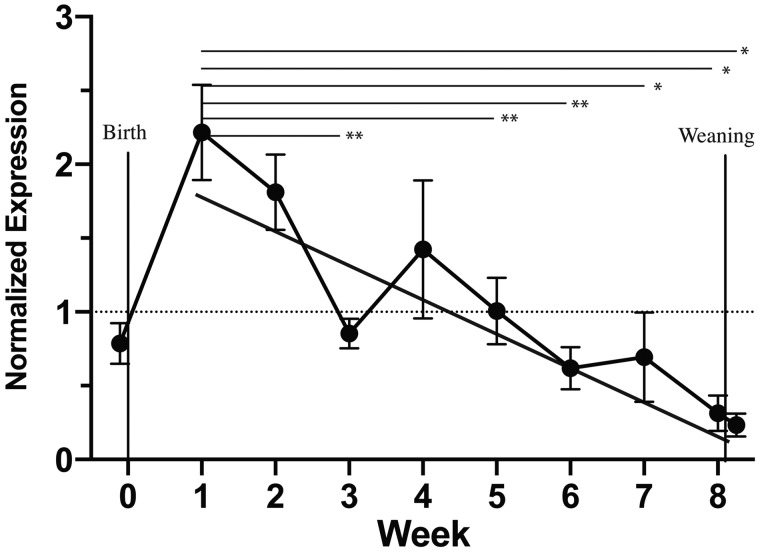
IL-16 is elevated early and declines throughout lactation. IL-16 abundance was highest in the first week postpartum and decreased linearly throughout weaning (slope=−0.2399, *y*-intercept=2.195, *r*^2^=0.848, and *P*=0.0004). *P<0.05, **P<0.01, ***P<0.001, ****P<0.0001. Dotted horizontal line denotes normalized expression = 1. Pooling as described in Supplementary Table S1.

Mammary development in eutherians has been associated with the presence of eosinophils (reviewed in Reed and Schwertfeger 2010). Eosinophils were also evident in opossum mammary early in the first postnatal week (Fig. 3). In P3 mammary sections, eosinophils were detected surrounding alveoli in early stages of development and were commonly found in the stroma surrounding developing alveoli. Eosinophils were only found in the first week and not in mammary tissues from weeks 2 to 7 (not shown). Since IL-16 has been shown to be chemotactic for eosinophils in other species (Rand et al. 1991), IL-16 transcript abundance in the opossum mammaries was investigated using qPCR. IL-16 transcripts were significantly elevated following parturition and decreased linearly through weaning (Fig. 4), correlating well with the presence of eosinophils and their absence at later time points (Fig. 3).

**Fig. 5:**
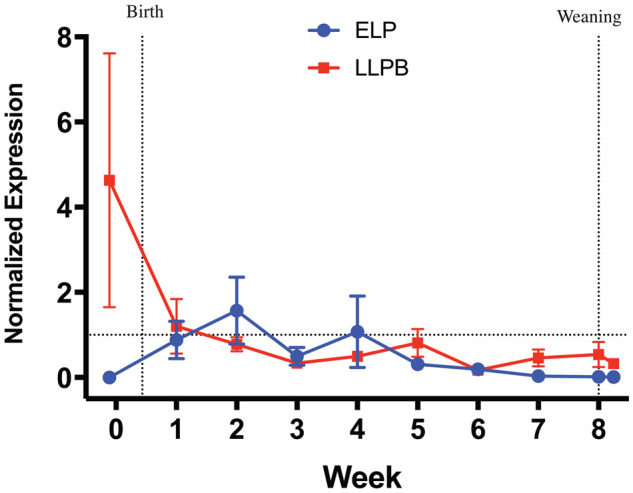
Early lactation protein (ELP) and late lactation protein (LLP) transcript abundance within the opossum mammaries. Transcript abundance of ELP (blue circles) and LLP-B (red squares) by week. Annotations as in Fig. 4. Minimal transcripts for ELP and LLP-B were detected during active lactation. Elevated transcription of LLP-B was only evident prior to birth, however inter-individual variance in expression exists.

To establish if the presence of ELP and LLP-B correlates with morphological change in the opossum mammaries, the abundance of transcripts encoded by these genes was determined. No ELP transcripts were detected in mammaries prior to birth (Fig. 5). Low levels of transcripts were first detected late in postpartum week 1 with minor peaks in abundance at weeks 2 and 4; however, these increases were not statistically different from zero (Fig. 5 andSupplementary Fig. 1A). LLP-B transcripts were detected in elevated abundance in the last 24 h prior to parturition and declined to low levels throughout postnatal lactation (Fig. 5 andSupplementary Fig. 1B). Other than the peak of LLP-B transcripts late in pregnancy, neither ELP nor LLP-B were detected in great abundance at any time point across active lactation in the opossum mammaries.

**Fig. 6:**
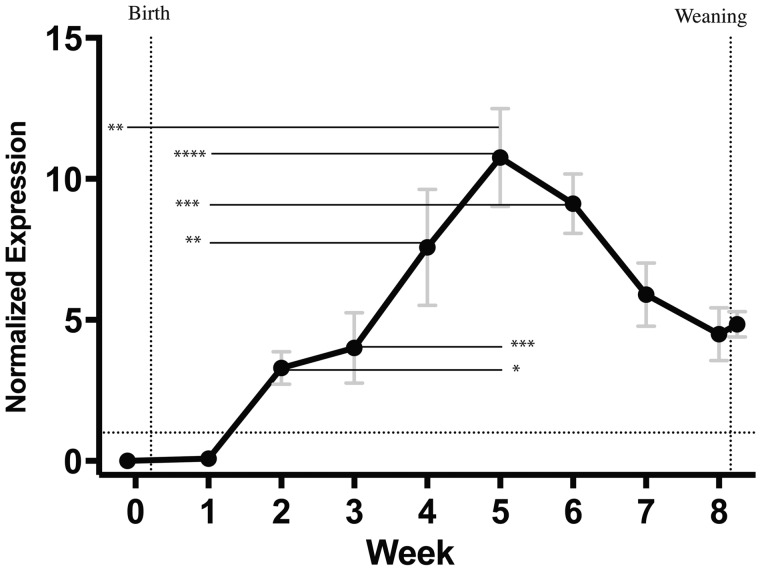
Whey acidic protein (WAP) transcript abundance within the opossum mammaries throughout lactation. Transcript abundance of WAP by week. Transcripts for WAP were first detected week 2 with peak abundance week 5. Elevated abundance was detected throughout the remainder of lactation. *P<0.05, **P<0.01, ***P<0.001, ****P<0.0001. Dotted horizontal line denotes normalized expression = 1. Pooling as described in Supplementary Table S1.

Another protein used to describe changes in nutritional composition in Australasian marsupials is WAP (Demmer et al. 2001; Nicholas et al. 2001; Kuy et al. 2007). WAP transcripts were not detected in opossum mammaries until the second week of lactation (P10) and followed a bell-shaped pattern from weeks 2 to 8 with peak abundance week 5 (Fig. 6 andSupplementary Fig. 1C). Despite declining abundance during the latter half of lactation WAP transcription remained elevated, relative to reference gene expression, throughout the duration of lactation and following removal of offspring for weaning at P56.

## Discussion

Mammaries were an evolutionary innovation unique to the lineage of synapsids from which mammals arose (Oftedal 2012). The evolution of mammaries from apocrine-like glands appears to have involved coordination with components of the immune system (Reed and Schwertfeger 2010; Oftedal 2012; Fehrenkamp and Miller 2019). This coordination is not necessarily surprising given the role milk plays in the transmission of maternal immunity to mammalian neonates (reviewed in Brandtzaeg 2010). A better understanding of the origins of the mammaries and the role cells of the immune system play can be achieved through comparative studies to discover conserved and divergent features.

The overall structure of the mature opossum mammary is very similar to that in eutherians. It is the state of mammary development at the time of parturition and the onset of milk production where the opossum differs. In eutherians remodeling of mammary tissue occurs during pregnancy in preparation for milk production (Djonov et al. 2001; Macias and Hinck 2012). Late in eutherian pregnancy, secondary and tertiary ductal branching is present and alveoli encompass the majority of the fat pad; however, there is little milk production *per se* (Djonov et al. 2001). In contrast, in the opossum there is neither mature alveoli nor ducts in the mammaries at the time of parturition. This is in spite of the immediate onset of milk production following birth. Furthermore, complex ductal branching and alveolar maturity were not evident in opossum mammaries until week 5 of the 8-week lactation period (Figs. 1 and 2). In other words, the developmental state of eutherian mammaries during active lactation is not achieved until the latter half of active lactation in the opossum.

We found that the presence of eosinophils in mammary tissue during early remodeling is conserved between eutherians and marsupials. Eosinophils were present in opossum mammary tissue in the first postnatal week, but not at later time points, correlating well with when remodeling of the tissue is greatest, as well as when IL-16 transcripts were most abundant. This is consistent with IL-16’s role in eosinophil chemotaxis as reported elsewhere (Rand et al. 1991). Eosinophils are believed to be fundamental in developing eutherian mammary tissue however their exact role remains unknown (Reed and Schwertfeger 2010). It is possible that secretions from eosinophils, or cytokines are transferred to the milk to serve as immunomodulators within the developing neonatal gut. It is unknown whether eosinophil effector molecules are secreted into the milk or absorbed by the neonate in this species. In this sense it is possible that eosinophil presence during early active lactation is serving as an additional layer of passive immune transfer in marsupials that is not seen in eutherian species.

Previously we described T cell infiltration in the developing opossum mammaries. γδ T cells were the predominant T cell type and were found in greater abundance in the first 5 weeks of lactation when mammary remodeling was greatest, consistent with reports in developing eutherian mammaries (Fehrenkamp and Miller 2019). Combined with the esosinophil results presented here, an early co-opting of immune cells in the remodeling of developing mammary tissue from an evolutionary perspective is supported.

The time line of mammary remodeling in the opossum is compressed compared with reports in other marsupial species. Enlarged alveoli and less dense stroma were not reported in the tammar wallaby until 37 weeks into active lactation compared with 5 weeks in the opossum (Findlay 1982; Nicholas 1988). This correlates with differences in developmental growth rate and time to weaning between opossums and the larger macropod marsupials; opossums are typically weaned at 56 days compared with 350 days in the tammar wallaby (Nicholas et al. 1997; Vandeberg and Robinson 1997).

**Fig. 7:**
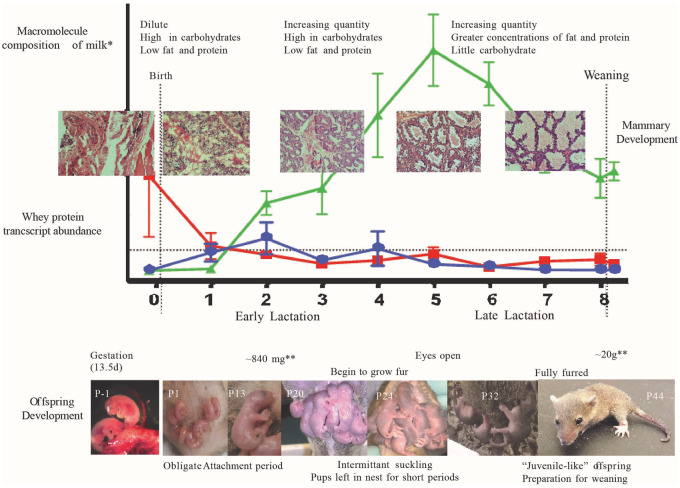
Morphological remodeling correlates with offspring development and nutritional composition. Histological photos are representative images from gestation E3, P3 (week 1), P16 (week 3), P32 (week 5), and P44 (week 7) in order of appearance from left to right. Morphological development of the mammaries correlates with previously described changes in composition and increased quantity of milk production (*adapted from Green et al. 1991). Histological maturity (week 5) correlates with peak transcript abundance of WAP (green triangles). ELP (blue circles) and LLP-B (red squares) do not appear to play a major role in opossum lactation. Mammary maturity (week 5) also correlates with major developmental milestones in offspring. Offspring developmental images are from the last 24 h of pregnancy (E13), P1 (week 1), P13 (week 2), P20 (week 3), P24 (4), P32 (week 5), and P44 (week 7). **Offspring weights adapted from Vandeberg and Robinson (1997).

Opossum mammary maturation correlates with offspring development. At postnatal week 5 when the mammaries have reached their peak development, neonates are covered in fur, have opened their eyes, and are beginning to seek solid food but are not fully independent (VandeBerg and Williams-Blangero 2010; BDF observations; Fig. 7). Also, by week 5 opossum neonates have a more developed adaptive immune system and appear to be becoming less dependent on maternal immunity (Parra et al. 2009; Wang et al. 2012). Indeed, at this time there is a decrease in transcription of *FcRN*, the receptor that transfers maternal IgG, in both the mammaries and the neonatal intestine, signaling a decrease in the transport of maternal antibodies as the pup matures (Fehrenkamp et al. 2018).

**Fig. 8:**
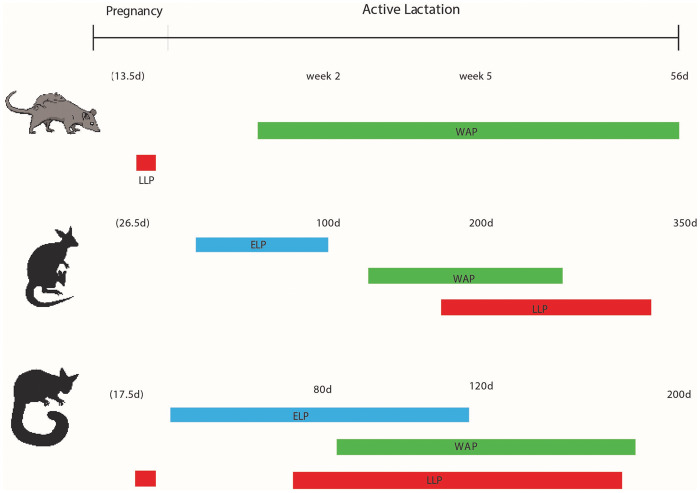
Schematic comparison of phase specific gene expression across lactation in three marsupials. Stage specific whey protein transcript abundance is represented in opossum, *M. domestica* (top panel), tammar wallaby, *Macropus eugenii* (middle panel), and Australian common brushtail possum, *Trichosurus vulpecula* (bottom panel). Relative timing of detected elevated expression of ELP (blue bar), LLP (red bar), and WAP (green bar) are represented. Expression in *M. eugenii* and *T. vulpecula* was adapted from Joss et al. (2009) and Trott et al. (2002), respectively. Research in *T. vulpecula* did not distinguish between LLP-A and LLP-B expression. Only the LLP-B homolog exists in the opossum and is reported here. Schematic LLP expression in *M. eugenii* is representative of both paralogs.

Histological maturation of opossum mammaries also correlates with nutritional changes in the milk produced. WAP transcripts were detected beginning week 2 with greatest abundance at week 5. It is noteworthy that this peak abundance correlates with mammary maturity. Based on transcript abundance WAP would likely be the predominate protein within the milk from early-middle lactation through weaning (Fig. 7). Transcript abundance also correlates well with previous reports of general protein abundance in opossum milk (Green et al. 1991). WAP is the whey-associated protein with the greatest conservation of expression among marsupials. WAP has been described as a “mid–late” lactation protein in Australian marsupials (Simpson et al. 2000; Demmer et al. 2001; Kuy et al. 2007; Joss et al. 2009). WAP transcripts were detected throughout lactation well into weaning in the opossum (Figs. 6 and 7). This is in contrast to the tammar wallaby and brushtail possum where WAP transcripts decline to undetectable by weaning (Fig. 8;Demmer et al. 2001; Kuy et al. 2007; Joss et al. 2009). This may be due to the longer lactation period of the larger marsupial species.

In Australian species, two paralogs of LLP have been detected, LLP-A and LLP-B (Trott et al. 2002; Joss et al. 2009; Hewavisenti et al. 2016). The opossum genome contains a single LLP gene that appears to be an ortholog to LLP-B in Australian species (Joss et al. 2009; Hewavisenti et al. 2016). When combined with the results from two Australian marsupial species, we find three different patterns of LLP expression (Fig. 8). In the opossum LLP transcripts were only detected early, prior to birth. In the tammar wallaby it has only been found later in lactation, hence the origins of the name: LLP. Lastly, in the brushtail possum, it is found both prior to birth and in late lactation (Demmer et al. 2001). Based on the pattern of expression in tammar wallaby, LLP has been speculated to play a role in the transition to an herbivore diet (Nicholas et al. 1987). While this is certainly a possibility in the herbivorous marsupials, it does not appear to be the case in the opossum, which are an omnivorous species. Likewise, it is unknown if there is a biological significance to the Australian marsupials having two paralogous copies of the LLP gene, nor what role LLP might play surrounding parturition in the opossum and brushtail possum.

Among the whey-associated proteins, opossums differ the most from other marsupials in the presence of ELP (Fig. 8). In tammar wallabies and brushtail possums this protein is found early in lactation and declines with the appearance of LLP (Trott et al. 2002; Joss et al. 2009). Opossums have an ELP ortholog, however, transcript abundance was low throughout lactation. The role of ELP remains unknown making it difficult to speculate on the species differences.

## Conclusion

A feature of marsupial development is that many ontogenic stages that occur during the prenatal period in eutherians are postnatal in marsupials. This appears reiterated in maternal mammary development where the opossum mammaries do not reach until later in the postnatal period a level of morphological development equivalent to that of eutherians at birth. Despite this difference a number of features are conserved among marsupials and between marsupials and eutherians. Noteworthy is the presence of immune cells such as eosinophils and T cells during maturation of the lactating mammary. These results support such features as ancient and likely present at the origin of mammary tissue as a defining characteristic of mammals.

## Author contributions

B.D.F. carried out the tissue collection, molecular lab work, histological work, data analysis, participated in the design of the study, and drafted the manuscript. R.D.M. conceived of the study, designed the study, coordinated the study, and helped draft the manuscript. Both authors gave the final approval for publication.

## Supplementary Material

obz036_Supplementary_Data
