# Supplementary material for: Opossum Mammary Maturation as It Relates to Immune Cell Infiltration and Nutritional Gene Transcription
Source: Integr Org Biol. 2019 Dec 30;2(1):obz036. doi: 10.1093/iob/obz036 (PMC7291930; doi:10.1093/iob/obz036)
Supplement: obz036_Supplementary_Data [file obz036_supplementary_data.zip › DevSuppTable2.pdf]

Gene symbol	Gene name	Ensembl gene ID	Target use	Primer Sequence	Primer location	Annealing Temperature (°C)	Amplicon length (cDNA)	Primer efficiency	Calibration curve slope	Calibration Curve y-intercept	Calibration curve r^2
ACTR2	Actin Related Protein 2 Homolog	ENSMODG00000003364	Reference	F:TGATCAACGTGGAAGGAGTG<br>R:ATCCTCCAGAAAGCACGATG	Exon 1<br>Exon 2	63	111	101.4%	-3.290	42.963	0.997
SDHA	Succinate dehydrogenase	ENSMODG00000000624	Reference	F: AAGAGGCTGTGGTCCTGAAA<br>R: CTTGGTGACATCCACACCAG	Exon 2<br>Exon 3	63	130	107.3%	-3.159	43.118	0.993
WAP	Whey Acidic Protein	ENSMODG00000010540	Target	F: GCTGCAGCTGGATGTGTAAG<br>R: ACAGGTTCGACCACAAGAGC	Exon 2<br>Exon 3	63	164	109.7%	-3.109	45.49	0.996
ELP	Early Lactation Protein	ENSMODG00000017492	Target	F: CAACTGCAAGGTCTTGTGAGC<br>R: TCCCCACAAATCAGAGGTTC	Exon 2<br>Exon 3	63	130	100.5%	-3.311	54.207	0.979
LLPB	Late Lactation Protein B	ENSMODG00000017492	Target	F: TCAGAGTGGCCAAACTTGTG<br>R:GCCTACTCAGGAGTGCAAGAC	Exon 4<br>Exon 5	63	145	101%	-3.299	51.339	0.773
IL-16	Interleukin-16	ENSMODG00000001265	Target	F: TTGTGCTGATGAAAGGCCAG<br>R: GTAACCTTCCATCAGCAGCAG	Exon 7<br>Exon 8	65	135	95.8%	-3.427	47.451	0.988
