# Supplementary material for: Opossum Mammary Maturation as It Relates to Immune Cell Infiltration and Nutritional Gene Transcription
Source: Integr Org Biol. 2019 Dec 30;2(1):obz036. doi: 10.1093/iob/obz036 (PMC7291930; doi:10.1093/iob/obz036)
Supplement: obz036_Supplementary_Data [file obz036_supplementary_data.zip › Dev_SuppTable1.pdf]

Supplementary Table 1: Summary of time points of mammary collection, number of biological replicates and associated method of preservation.

Week	Time points included (# of biological replicates)	Tissues Used in Histological Examinations	Tissue Used in Gene Expression Analyses
0	E3 (1)	X	
	E14 (3)	X	X
1	P1 (3)		X
	P2 (3)		X
	P3 (7)	X	X
	P5 (3)		X
	P7 (3)	X	X
2	P10 (3)	X	X
	P13 (3)	X	X
3	P16 (3)		X
	P17 (6)	X	X
	P20 (3)		X
4	P22 (3)		X
	P26 (3)	X	X
5	P31 (3)		X
	P32 (9)		X
	P33 (3)	X	X
6	P36 (3)	X	X
	P38 (3)		X
7	P44 (6)	X	X
8	P52 (3)		X
Post weaning	24-48 hours after removal of offspring at P56 (3)		X
