# Supplementary figures and images for: Opossum Mammary Maturation as It Relates to Immune Cell Infiltration and Nutritional Gene Transcription
Source: Integr Org Biol. 2019 Dec 30;2(1):obz036. doi: 10.1093/iob/obz036 (PMC7291930; doi:10.1093/iob/obz036)

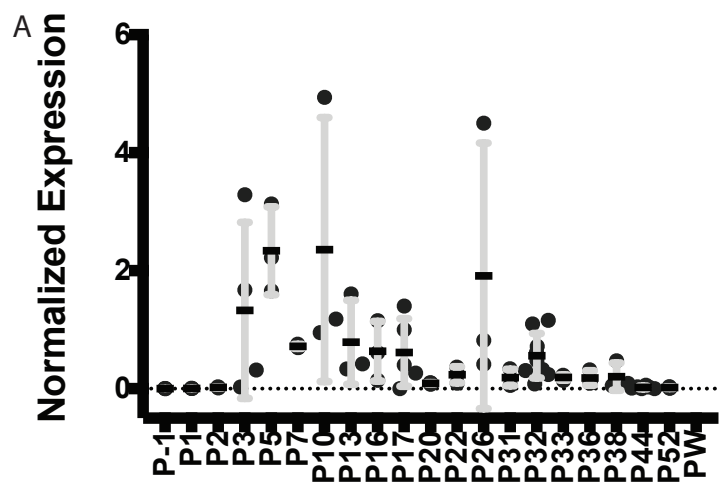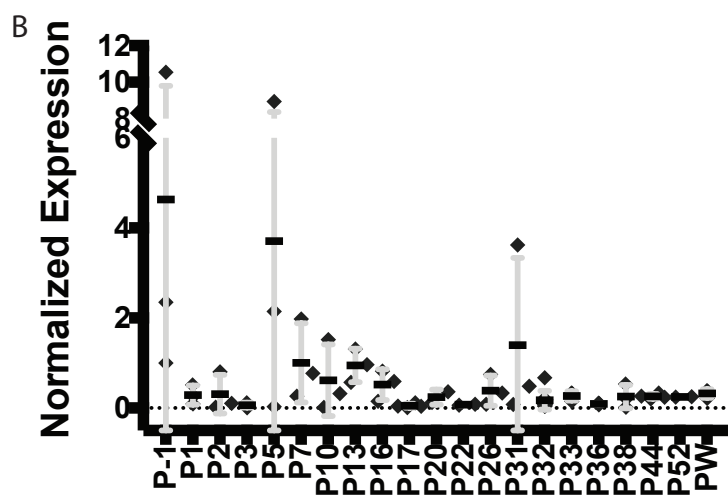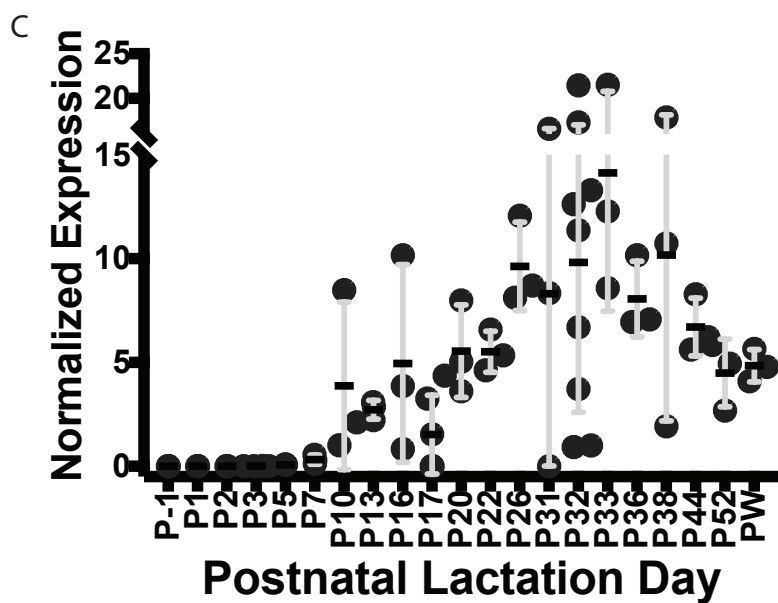

Supplement: obz036_Supplementary_Data [file obz036_supplementary_data.zip › Dev_Supp_Fig1.pdf]
